# Self-reported stressors among patients with Exhaustion Disorder: an exploratory study of patient records

**DOI:** 10.1186/1471-244X-14-66

**Published:** 2014-03-05

**Authors:** Karin Hasselberg, Ingibjörg H Jonsdottir, Susanne Ellbin, Katrin Skagert

**Affiliations:** 1 The Institute of Stress Medicine, Carl Skottsbergs gata 22B, SE 413 19 Gothenburg, Sweden

**Keywords:** Burnout, Exhaustion, Stressors, Non-work stressors, Work stressors, Mixed methods

## Abstract

**Background:**

Several researchers imply that both work-related and non-work-related stress exposure are likely to contribute to stress-related mental illness. Yet empirical studies investigating both domains seem to be limited, particularly in a clinical population. The purpose of this study was to a) explore which stressors (non-work and work-related) are reported as important for the onset of illness by patients seeking medical care for stress-related exhaustion and b) explore the prevalence of each stressor and examine whether the pattern differs between men and women.

**Methods:**

This is an exploratory mixed method study, comprising patients at a specialist outpatient stress clinic. Information from medical records of 20 patients was initially used in a first qualitative step to construct the instrument, using a combination of a conventional content analysis and a directed content analysis. In the second phase patient records from 50 men and 50 women were selected and coded in accordance with the coding instrument. Frequency statistics were calculated for all stressors.

**Results:**

A total of 24 categories of stressors (11 related to work and 13 related to private life) were identified in the first qualitative step. A median of four stressors, usually both work and non-work-related was reported by the patients. The most common stressors were 1) quantitative demands at work, 2) private relational conflicts and 3) emotional demands at work.

**Conclusions:**

Work demands are, by far, the most prevalent stressor, followed by relational problems in private life. The pattern was similar for women and men, with a slight difference in the distribution between work and non-work stressors. Men and women also show similar patterns when comparing the occurrence of each stressor. Slight differences were seen, in particular with regard to managerial responsibility that was reported by 6% of the women compared to 36% of the men. One important practical implication of this study is that patients with stress-related exhaustion often have a long period of impaired ability at work. Successful prevention at the workplace is thus of great importance. However, it is equally important to discuss how society can support individuals such as single parents or couples with relational conflicts.

## Background

Stress-related illness is a major concern in many countries [1-3] and years of extensive research have concluded that exposure to psychosocial stress is an important risk factor for physical [4,5] as well as for mental [6,7] symptoms. Psychosocial stress is thought to be one of the main reasons for the increase in mental illness seen during the past decade, as well as the increases in long-term sickness absence due to mental health problems [1,8,9].

It has been known for decades that stressful life events can affect our health and scientific research in this area was greatly expanded in the early 60s when Holmes and Rahe studied the relationship between stressful life events and illness. Their research resulted in the widely used Social Readjustment Rating Scale (SRRS) [10]. The scale consists of 42 life events, ranging from death of spouse to celebrating Christmas, and six of them are directly related to work [10]. Many years have passed since the scale was developed and we can assume that the character and the perception of stress exposure, related to both work and family life, has changed and hence that the rating of each item in the scale poorly reflects the stress exposure in present-day society. In a fairly recent quantitative revision of the weights within the scale, the SRRS is criticized as being a mixture of life events (stressors) and outcomes due to a stressor [11]. Furthermore, the modern life situation most certainly includes some other stressors that were not included in a scale that was developed 50 years ago. Thus, SRRS might not be suitable to assess thoroughly today’s stress exposure.

The literature regarding psychological and mental outcomes of stress exposure and prolonged stress consists of several partly overlapping concepts. The most widely used and internationally recognized term is burnout, originally defined by Maslach [12]. This concept is not coherent and the theoretical basis of burnout varies depended on the self-report instruments used to assess the condition [13]. The concept of burnout has been useful in studying mental and physical health problems in the working population [14,15]. However, it is not a clinically defined diagnosis and thus not very useful in clinical practice.

In 2003 the National Board of Health and Welfare in Sweden proposed diagnostic criteria for Exhaustion Disorder (ED), in order to identify a more homogenous clinical group of patients suffering from pronounced mental and physical exhaustion due to prolonged stress [16]. In the International Classification of Diseases and Related Health Problems (ICD-10) the diagnosis of stress-related exhaustion was assigned the code F43.8A [17]. ED and burnout are close entities and an overlap of 93% is seen between ED and self-reported burnout [16]. An important diagnostic criterion for ED is the identification of defined stressors, which are assumed to be the cause of the present exhaustion. The stressors should have been present for at least six months and can be either work-related or non-work-related [16].

In spite of extensive research on the relationship between mental illness and stress, most research has, until today, focused mainly on work-related stress exposure [16] and indeed the concept of burnout is based on the fact that the exposure is due to work-related factors [12]. This is also evident in the two most influential occupational health theories on how psychosocial stress contributes to a mental health problems, namely the effort-reward-imbalance model [18] and the demand-control-support model [19,20]. The Copenhagen Psychosocial questionnaire is theoretically based on these models, and attempts to cover stressors and resources that are of importance for the psychosocial work environment [21]. The questionnaire includes scales regarding demands and social relations at work, conflicts at the workplace and work-family/family-work conflict, but does not measure non-work stressors [21].

Since most research on the determinants of stress-related mental illness is concerned with work-related stress exposure, research on non-work related stress exposure is important as this exposure has been proposed by several researcher to be equally important [6,7,22].

A small number of studies have identified associations between stress-related mental problems and non-work stressors, such as recent life events [7], caring responsibilities [23,24], work-to-family conflict [25] and lack of social support [26]. Even though multi-factorial causation is indicated, most of these studies focus on only one type of non-work stressor and, as other research groups have pointed out [6,22], the conceptualization of non-work stressors is poor and measures of non-working stressors are considered insufficient in existing studies.

In a review by Beauregard et al. [22], some evidence was found for an association between non-work stressors and workers’ mental health, but the authors conclude that the evidence of non-work determinants on mental health is insufficient due to weak conceptualizations and shortage of studies. Two studies have found independent effects of both work and non-work stressors on common mental disorders (CMD, i.e. mood and anxiety disorders) [6,27], indicating that non-work stressors are associated with CMD regardless of co-existing work stressors and vice versa. Clark et al. [6] concluded that both types of stressors are important in the understanding of CMD and that interventions aimed at reducing psychosocial stress should address both work and non-work stressors.

Thus, even though several researchers have put forth the hypothesis that stress exposure at work and outside of work are likely to contribute to stress-related mental illness, the empirical work on this seems to be very limited [22,28]. Most studies investigate either stressors within the work domain [29,30] or, if stressors outside of work are studied, only a limited number of stressors are included [26,31]. Further, very few studies of both work and non-work stressors in a clinical population are available and to our knowledge no such study exists on the population of patients with stress-related exhaustion.

Furthermore, since the research on the contribution of non-work stressors to stress-related mental illness is scarce, gender aspects in this area are even less explored and the findings that exist are inconsistent. Mental health problems are more commonly reported by women [27] but there is as yet no comprehensive explanation of this [32]. Even though some studies have found gender differences in stress exposure, for example social roles [23] and caring responsibilities [31], most studies have failed to support gender differences when it comes to the association between stressors and mental health outcomes [6,23,26,33].

The present study was conducted in order to explore which stressor or stressors patients seeking medical care for stress-related exhaustion report to be the cause of the onset of their illness. As basically all previous literature focuses on a limited number of predefined stressors, this is the first study exploring all possible stressors, regardless of origin (non-work or work-related), reported by this clinical population. Hence, the purpose of the present study was to a) explore the stressors (non-work and work-related) reported by patients diagnosed with ED, b) to explore the prevalence of each stressor and c) to investigate whether men and women differ in regard to the types of stressors reported.

## Methods

### Design

This is an exploratory mixed method study [34], using a qualitative approach followed by a quantitative summary of patients’ medical records. The initial step was performed to gain a comprehensive understanding of reported stressors, in order to construct a coding tool to quantitatively collect information about stressors in a larger sample.

### Setting and participants

This study comprises patients referred to a specialist outpatient clinic in Gothenburg, Sweden, exclusively treating patients with stress-related exhaustion. Information from medical records of 20 patients was initially used to construct the coding instrument. Data extracted from medical records from 100 patients (50 women and 50 men) were then included in the main data analysis. All patients are referred to the clinic by primary health-care centres and occupational health service centres and the referral criteria are stress-related exhaustion with no apparent somatic disorder or abuse that could explain the exhaustion, and a maximum duration of ongoing sick-leave of six months. All patients included in this study fulfilled the basic referral criteria to the clinic, these being 1) working age (18–64 years old), 2) not been on sick-leave more than 6 months and 3) no known co-morbid diagnosis that can explain the patient’s problems (except for depression and anxiety). An additional inclusion criterion for this present study was that the patients should fulfil the diagnostic criteria for Exhaustion Disorder (ED) (Table 1). Patient records from 50 men and 50 women were selected through backwards consecutive inclusion, starting from September 2011. Patients included in the present study had their first visit to the specialist clinic in the years between August 2007 and August 2011. A senior physician at the clinic carried out a diagnostic procedure on the patients, obtaining an extended anamnesis with a clinical examination. The DSM-IV based instrument PRIME-MD (one-page patient questionnaire) was filled in by the patient and the results were used as a support in the diagnosis of depression and/anxiety. Whenever necessary this included a structured interview to identify any presence of mood and/or anxiety disorders. To be diagnosed with ED it is required that the physician, together with the patient, is able to identify one or more stressors that have been present for at least six months, during which time the symptoms have developed. The criterion does not specify the type or intensity of the stress exposure but it is implicit that it should be significant enough to provoke the stress symptoms. The physician goes through the complete ED diagnostic criteria (Table 1) with the patient. A and B are obligatory criteria, as is the presence of at least four of six symptoms listed under C. Also, the condition should cause significant distress and/or impairment of important areas of functioning (D) and no symptoms should be due to direct physiological effects of a substance or a general medical condition (E). Finally, if the patient does meet the criteria for major depressive disorder, dysthymic disorder or generalised anxiety disorder, these diagnoses are set first and ED is set as a co-morbid condition.

**Table 1:** Diagnostic criteria for stress-related exhaustion disorder as proposed by the Swedish National Board of Health and Welfare 2005

**Diagnostic criteria for exhaustion disorders**		
**A**	Physical and mental symptoms of exhaustion with minimum two weeks duration. The symptoms have developed in response to one or more identifiable stressors which have been present for at least 6 months.	
**B**	Markedly reduced mental energy, which is manifested by reduced initiative, lack of endurance, or increase of time needed for recovery after mental efforts.	
**C**	At least four of the following symptoms have been present most of the day, nearly every day, during the same 2 week period:	
	*1 Persistent complaints of impaired memory.*	
	*2 Markedly reduced capacity to tolerate demands or to work under time pressure.*	
	*3 Emotional instability or irritability.*	
	*4 Insomnia or hypersomnia.*	
	*5 Persistent complaints of physical weakness or fatigue.*	
	*6 Physical symptoms such as muscular pain, chest pain, palpitations, gastrointestinal problems, vertigo or increased sensitivity to sounds.*	
**D**	The symptoms cause clinically significant distress or impairment in social, occupational or other important areas of functioning.	
**E**	The symptoms are not due to the direct physiological effects of a substance (e.g. a drug of abuse, a medication) or a general medical condition (e.g. hypothyroidism, diabetes, infectious disease).	
**F**	The stress-related disorder does not meet the criteria for major depressive disorder, dysthymic disorder or generalized anxiety disorder.	

A written informed consent to use the journal records for research purposes was obtained from all participants prior to inclusion in this study. The study was approved by the Regional Ethical Review Board in Gothenburg and conducted in accordance with the Declaration of Helsinki. All the results are presented at group level and no individuals are possible to identify in the results.

### Procedure and analysis

Pre-collected information from the medical records at the clinic was used in this study. During the first visit to the clinic, stressors were identified by the physician together with the patients as a part of the diagnostic procedure. Only information from the first visit to a physician at the clinic was included in the study. The first visit to a physician was approximately 90 minutes in length, semi-structured and covered the patient’s medical and social history. Stressors were identified by a procedure where the physician exemplifies different types of stressors (physical/environmental, emotional and social, both work-related and non-work-related) and then asks the patient about the stressors that he/she experiences. The diagnosis of ED and other relevant diagnoses, such as depression and anxiety disorders, are established by the end of the appointment. Immediately after the appointment, the physician wrote a detailed summary of what had emerged.

#### Construction of the coding instrument (step 1)

In the initial phase 20 patient records from the first visit to the clinic were extracted for analysis. A strategic selection of patients (10 women and 10 men) was made with the intention of getting dispersion in terms of age and family composition, i.e. single/in a relationship, children/no children. The mean age for the group used to construct the instrument was 47 years (range 29–63 years). The majority were married or had a partner (n = 17), while only seven had children who still lived at home and three of the patients did not have any children. The remaining (n = 10) reported that they had adult children.

The patient-records were analysed in accordance with Content Analysis [35] with the aim of identifying what stressors are reported as related to the onset of the exhaustion. Content analysis is defined by Hsieh & Shannon [36] as “a research method for the subjective interpretation of the content of a text through a systematic classification process of coding and identifying themes or patterns”. A combination of a conventional content analysis and a directed content analysis was used in this study [36]. Conventional in the sense that categories were not decided beforehand, but derived from the data. Directed since the coders had a prior understanding of categories identified by other researchers [10,21], which were considered in, but did not control, the making of categories. The partitioning of categories into work and non-work stressors was decided beforehand, which also contributed to a more directed approach of content analysis. Only manifest content was coded in this analysis. Each patient record was read through and all information identified as a potential stressor related to the present exhaustion was highlighted. Highlighted text was then labelled with different codes and the codes for all 20 records were then grouped together into categories of stressors. This was done by two coders independently and categories were then compared and discussed and a merged template of categories was formed. The coders were the last-named author, a PhD and experienced qualitative researcher, and the first author, a clinical psychologist with training in qualitative research and content analysis. The initial coding when developing the instrument was thus validated by the two independent coders, and no differences were found between the coding when compared. When 20 patient records had been analysed both coders found the material to be saturated and no more patients were included in this phase, a procedure that is recommended for Content Analysis [35]. In total, 24 categories of stressors were identified, of which 11 were related to work and 13 were non-work-related.

#### Main data analysis (step 2)

In the second phase patient records from 50 men and 50 women were selected through backwards consecutive inclusion. A description of the sample is found in Table 2. The mean age for this sample was 42.6 years, with no significant difference between men (42.2 years) and women (43.0 years; *p* = 0.116). Being married or living with a partner was more common among men and being employed in the public sector (most schools, elderly care and hospitals are public in Sweden) was more common among women.

**Table 2:** Descriptive of the 100 patients included in the quantitative analysis

***Characteristics***	***Women***	***Men***	***Total***	***P-value***
	***N = 50***	***N = 50***	***N = 100***	
	**n (%)**	**n (%)**	**n (%)**	
Marital status				.050
-Married/partner	39 *(78)*	46 *(92)*	85 *(85)*	
-Single or other	11 *(22)*	4 *(8)*	15 *(15)*	
Comorbidity				.297
-Depression	5 *(10)*	3 *(6)*	8 *(8)*	
-Anxiety	8 *(16)*	3 *(6)*	11 *(11)*	
-Depression and anxiety	37 *(74)*	42 *(84)*	79 *(79)*	
-No comorbidity	0 *(0)*	2 *(4)*	2 *(2)*	
Employer^1^				.049
-Public sector	20 *(51)*	15 *(31)*	35 *(40)*	
-Private sector	19 *(49)*	34 *(69)*	53 *(60)*	
Education^2,3^				.774
-Higher	33 *(67)*	31 *(65)*	64 *(66)*	
-Lower	16 *(33)*	17 *(35)*	33 *(34)*	

^1^Missing data for 11 women and 1 man

^2^Higher education is one year of university education or more.

^3^ Missing data for 1 woman and 2 men.

Each patient record was then coded in accordance with the coding instrument developed in step 1, so that each category of stressor that emerged in a patient’s record was coded once.

The coding during the main analysis of patient records (n = 100) was determined by one coder, but whenever an ambiguity arose this was discussed with the other researcher for a second opinion. This was the case for 13 of the 100 patient records. One patient was excluded during the coding procedure due to a disinclination to register his stress exposure in the medical record. In order to continue the consecutive inclusion procedure, this patient was replaced with another patient fulfilling the inclusion criteria.

Data from the coding procedure was inserted into SPSS version 19.0 together with information about gender, age, marital status and employment, collected at the patient’s first visit to the clinic. Frequency statistics were then calculated for all stressors, both for the whole sample as well as for the subgroups of women and men. Chi-square test was used to analyse the demographics variables presented in Table 2. The 95% confidence interval (CI) for difference in proportions (diff) was carried out to identify possible gender differences for each reported stressor.

## Results

### Identified categories of work-related and non-work-related stressors

The methodological development of the instrument (step 1) resulted in a total of 24 categories of stressors, of which 11 were related to work and 13 were non-work-related. All categories are presented in Tables 3 and 4 together with a short clarification of each category.

**Table 3:** Qualitative categories of work stressors

***Work stressors***	***Categories***	***Explanation***
	Quantitative demands	Including cognitive demands, workload, time pressure, overtime work, constantly being interrupted.
	Emotional demands	Including emotional or psychological demands at work such as having more responsibility than one can handle, not having enough competence for one’s assignments and demanding tasks such as care-giving tasks or being responsible for clients/students.
	Conflicts	Conflicts with co-workers, subordinates, managers and/or clients.
	Managerial responsibility	When patients reported being in some kind of leadership position and experienced this as stressful.
	Reorganisation	Stressors due to reorganisation of the workplace or a high turnover of colleagues.
	Deficient leadership	Stressors related to various lack of leadership, e.g. not experiencing enough support from managers or reporting having an unpredictable manager.
	Job insecurity	When experiencing an insecurity related to one’s employment, like working as a contractor or having a temporary employment. This category also included having been dismissed from work.
	Irregular working hours	Work hours reported as exhausting/wearing, such as night work, overtime, irregular working hours or long-distance commuting.
	Noisy work environment	Stressors associated with a loud or disturbing work environment.
	Discontent	When explicitly expressing that one is not happy or satisfied with their workplace in general as a specific stressor.
	Traumatic event at work	For example having a patient committing suicide (applies to those working in health care), lawsuits or being exposed in media.

**Table 4:** Qualitative categories of non-work stressors

***Non-work stressors***	***Categories***	***Explanation***
	Death of a family member	Loss of parent, sibling, grandparent, ex-partner or a sibling-in-law. Varying time has passed since the loss, but it is reported to be a stressor related to the current exhaustion. Some patients reported more than one loss as a reason, and one patient had experienced three losses that were reported as being related to his/her exhaustion.
	Caring for a family member	When caring for an adult or child in the family with 1) a somatic condition such as meningitis, diabetes, asthma or cancer, or 2) a mental/psychiatric condition such as ADHD (Attention-Deficit/Hyperactivity Disorder), Asperger’s Syndrome, depression, bipolar disorder, self-injurious or suicidal behaviours and acting out behaviours.
	Single parent	Having sole responsibility for one’s children.
	Relational conflicts	Conflicts with family members such as partner, child, parents, parents-in-law, ex-partners or siblings.
	Separation	When a separation was reported as a factor contributing to the current exhaustion. The separations were dated from two months up to ten years back in time.
	Change in family composition	Stressors related to gaining new family members or having a family member move out
	Worries about one’s health	Extensive worry about ones own health, in some cases due to having a hereditary fatal disease in the family.
	Personal injury or illness	Exceptional worries due to the patient suffering from chronic illness such as diabetes, herpes zoster, cervical cell changes, benign paroxysmal, urticaria, or suffering from the aftermath of a miscarriage or a fall trauma.
	Financial worries	For example financial worries due to being unemployed or having temporary jobs
	Residential stressor	Stressors related to a change in housing situation, i.e. moving or building a new house or a stressor related to having practical problems with one’s residence, i.e. repairs or construction work.
	Voluntary engagement in associations	Having an overload of voluntary engagements in associations, sports clubs etc.
	Legal matter	Legal conflict or similar ongoing process.
	Loneliness	Experiencing living alone/not having a partner as a stressor.

### Prevalence of reported stressors

The patients (n = 100) reported a median of four stressors and this did not differ by gender. A maximum of eight (n = 1) stressors and a minimum of one (n = 3) stressor was reported. A median of three work-related stressors and one non-work stressor was reported by the whole sample. This distribution differed slightly by gender as men reported a median of three work stressors and one non-work stressor as compared to women who reported a median of two work stressors and two non-work stressors.

As seen in Table 5, a fifth of the patients (n = 20) did not report any non-work stressor and thus only reported work-related stressors. Only four patients did not report any work-related stressor and thus only reported non-work-related stressors.

**Table 5:** Number of reported work and non-work stressors by the patients diagnosed with stress-related exhaustion

***Number of stressors***	***Work-related stressors***			***Non-work related stressors***		
	**Women (n = 50)**	**Men (n = 50)**	**Total (n = 100)**	**Women (n = 50)**	**Men (n = 50)**	**Total (n = 100)**
0	3	1	4	9	11	20
1	10	8	18	14	18	32
2	17	8	25	14	14	28
3	9	21	30	4	5	9
4	8	7	15	8	1	9
5	3	5	8	0	1	1
6	0	0	0	1	0	1

**Figure 1:**
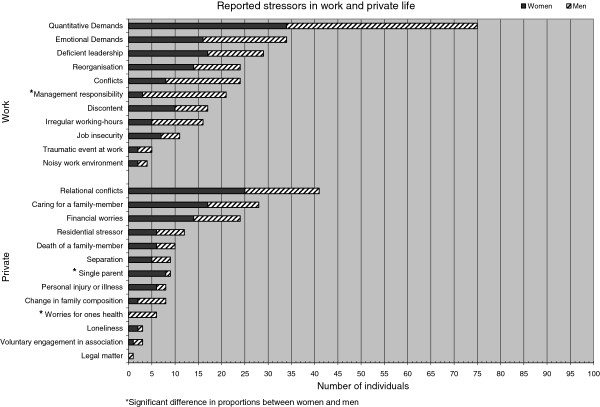
Number of patients with stress-related exhaustion, divided by women and men, reporting each stressor.

As seen in Figure 1, the most common stressors reported by the patients, regardless of whether they are work-related or non-work-related, were, 1) quantitative demands at work, 2) private relational conflicts and 3) emotional demands at work. The most common stressors among the non-work stressors were 1) relational conflicts, 2) caring for a family member and 3) financial worries, and this was irrespective of gender. In regard to work stressors the pattern differed somewhat by gender as the most commonly reported work stressors for men were 1) quantitative demands, 2) emotional demands and 3) managerial responsibility and conflicts at work (equally common), whereas women reported 1) quantitative demands, 2) deficient leadership and 3) emotional demands. Comparing the occurrence of each stressor, similar patterns were found for men and women, except for one work related stressor and two private life stressors. Thus, *Managerial responsibility* was reported by 6% of the women compared to 36% of the men (diff −30, 95% CI −44.4; −14.3). The two private life stressors that differ between men and women were *worries about one’s health*, reported by 0% of the women and 12% of the men (diff −12, 95% CI −23.8; −2.4) and being a *single parent*, reported by 16% of the women and 2% of the men (diff 14, 95% CI 2.6; 26.6).

## Discussion

The main finding in the present study was that patients with stress-related exhaustion report both work and non-work stressors as important for the onset of their illness. This result adds to the growing evidence that a combination of both work and non-work stress exposure has an impact on stress-related mental illness [6,22,28]. Almost all patients report more than one stressor and the average number of stressors reported by the patients was four stressors usually both work-related and non-work-related but with a slight domination of work-related stressors. Thus, it is uncommon that a single stressor is reported as the cause of stress-related mental exhaustion. Rather it is the combination of several stressors in people’s life as a whole that is reported to be the cause of their illness. Most patients experience these multiple stressors to be both work-related and non-work-related, but, with a fifth of the patients reporting only work-related stressors, it seems clear that stress-related exhaustion can be experienced solely due to stress exposure at work, but that it seldom appears to be a consequence of non-work-related stress only, or at least not in the patient sample included in this study.

Another important finding was that men and women reported the same amount of stressors, but with a slight difference in the distribution between work and non-work stressors. Women reported more non-work stressors than men, whereas men reported more work stressors. Previous studies have shown that the association between exposure to stressors and mental health problems is the same for women and men, indicating that women are affected by stressors in the same way as men are [6,23,26]. Women in general seem to be exposed to or to experience more non-work stressors than men [23,31] and domestic work and work-home interference have been indicated as more important for women [37-39].

The most commonly reported work stressors, besides the exposure to quantitative and emotional demands at work, seem to differ by gender in that men experienced more stress related to managerial responsibility and conflicts. Leadership issues therefore seem to be of relevance when it comes to the onset of stress-related mental illness. The gender difference in what management stressor was reported may be explained by the fact that it is more common for men to hold managerial positions than for women. The labour market is still strongly gender-segregated, both vertically and horizontally, with men and women working in different occupations and a subordination of women [40]. Others have also found associations between leadership, sickness absence and exhaustion, both in the means of the leadership style of one’s manager [41,42] as well as one’s own leadership style if in a leadership position [43]. Thus, the issue of leadership seems to be an important, though complex, issue/stressor of relevance to the onset of stress-related exhaustion.

At least one work stressor seems to be necessary for the onset of exhaustion for almost all patients, and demands at work are, by far; the most commonly reported stressor for both men and women in this study. This is consistent with previous research [30,44] and adds to the evidence of the importance of addressing demands at work in prevention and interventions aimed at reducing stress-related mental illness. The fact that work stressors are reported to a higher degree might as well be a result of what the patients choose to talk about at their first appointment with the physician. One could speculate that it is too difficult to talk about certain stressors and also that the patient forgets to share information on important stressors during the first visit. It might be of importance for physicians that encounter patients with stress-related mental health problems to ask more explicitly about non-work stressors when gathering information about the cause of illness, in order not to miss relevant information about the aetiology of the disorder for a specific patient.

Interestingly, the most commonly reported non-work stressor, relational conflict, seems to be similar for both women and men. The finding that relational conflicts in private life were reported by nearly half of the participants indicates that this is a highly important stressor that contributes to the development of stress-related mental problems. This is in line with previous research showing that negative aspects of relationships are the strongest predictors of psychological distress, for both men and women [26].

As in previous studies [23,27] the results show that care-giving responsibilities are a frequent stressors experienced by patients with stress-related mental illness, in this study expressed as a stressor of being a single parent and/or caring for a family member. Women do seem to report these stressors to a higher extent than men. These results highlight the importance of further research on non-work stressors as well as work stressors when attempting to explain the development of stress-related problems and particularly the gender aspects of stress-related mental problems and may contribute to further understanding of inequalities in mental health between men and women. Interventions aimed at reducing stress-related problems would also benefit from addressing these issues. Maybe it is necessary to reduce some of the responsibility for caring among women in order to reduce stress-related mental illness in this group, for example through better support from social services, health care services and day-care services for children.

Men seem to be slightly more likely to report worries about their own health and one explanation may be that men do not seek help until both mental and somatic symptoms are prominent, causing severe health problems in these patients. The nature of masculinity, i.e. being the “stronger” sex, does not “allow” physical or psychological weakness and men are not likely to seek help. Yet even if the prevalence of depression is higher among women, more men commit suicide [45]. However, due to the small size of the sample this result should be treated with caution and further studies are needed.

Although there is, to our knowledge, no previous research available studying the occurrence of stressors among patients with stress-related exhaustion, the main findings in the present study are in line with what has previously been suggested by other research groups [6,22], namely that both work and non-work stressors are important contributors to the onset of stress-related mental illness. Several of the events in SRRS seem to be outdated, since for example no patient reported either “wife beginning or ceasing work outside the home” nor “Celebrating Christmas” [10]. Yet, relational events or conflicts remain valid. The study highlights the importance of multiple (i.e. more than one) stressors in understanding the aetiology of stress-related mental problems and provides important information on what factors need to be considered in order prevent stress-related mental problems.

### Methodological considerations

There are several methodological considerations that should be raised. One major limitation is that the data analysed in this study is not collected by interviewing the patients. Data is obtained from the patient’s medical records written and interpreted by a physician. Thus, the data can only be considered for what it is, namely a summary of the patient’s own words based on the physician’s understanding and presentation of what the patient has expressed verbally. In an attempt to minimize a potential bias due to this procedure only patient records from the two most experienced physicians (out of 6 possible) at the clinic were used and we could not see any major difference in how they register stress exposure in the medical records.

The stressors were identified in a semi-structural manner and the two physicians work in a similar manner and both have a long experience of working with patients with exhaustion.

We cannot, however, entirely know whether they interpreted the information from the patients differently or whether e.g., age or sex (both physicians are women) or other factors could have influenced their interpretation of the patient’s history. The physician exemplifies different types of stressors (physical/environmental, emotional and social, both work-related and non-work-related) and then asks the patient to discuss the stressors that he/she experiences. This exemplification could be problematic as the patients could relate to them as if they were the stressors that they should report. The stressors reported by the patients are not identical with the given examples and the physicians endeavour to make sure to give the patient enough time to elaborate on their own stressful situation and not the examples given. This exemplification was done in similar manner and all the patients got the same examples to relate to. The ideal would be to interview the patients to get the primary source of the information, and it would be interesting to do this in future studies.

Another limitation of this study is related to the patient sample included. The patients referred to the clinic are on average highly educated with fairly high socioeconomic status. Thus the results from this study cannot be easily applied to other groups, e.g. with lower socioeconomic status or unemployed individuals. It is plausible that these groups would report other stressors to be important such as, possibly, financial worries. It is thus important in further studies to explore the stressors in other groups. The results from this paper give us an important knowledge of what kind of stress load is involved in the development of stress-related exhaustion. We cannot, however, conclude that these particular stressors will always lead to stress-related illness. Stress exposure is perceived differently by different individuals and in different contexts, and there are several other factors that contribute to the complexity of development of stress-related symptoms. These include both resilient and vulnerability factors such as personality, social support, self-esteem, lifestyle, genetics and socioeconomic status.

In this study, the calculation of reported stressors is based on qualitative analysis. We did not weight the different stressors or differentiate between long-term stressors and more acute ones, but as Lantz et al. [28] have pointed out, both types of stressors have been suggested to have an impact on stress reactions and psychological distress. Furthermore, the purpose of this study was not to investigate how stressors are associated with negative health outcome, but which stressors are considered to be of importance for the patient’s situation. Thus, some of the stressors found in this study might have been long-lasting, whereas others might have made one limited appearance; but all were considered equally important since we were trying to explore the patient’s subjective experience of which stressors were important for the onset of their illness. An additional methodological consideration is that the aim of this study was not to construct a new instrument. The purpose of our coding tool was merely to code and collect possible stressors in patients with stress-related exhaustion.

## Conclusions

Non-work-related stressors are nearly as prevalent as work-related stressors among patients with stress-related exhaustion. Work demands are, by far, the most prevalent stressor, followed by relational problems in private life. Future studies should thus investigate both types of stressors as factors contributing to stress-related mental problems. Women and men do not differ greatly in which stressors are reported. Women tend to report more non-work-related stressors and men relate their problems to work-related stressors. Otherwise, among individuals with quite high educational levels, the stress exposure involved in the onset of exhaustion does not seem to be gender-specific. The result of this study has some implications regarding prevention of stress-related mental health problems. Patients fulfilling the diagnostic criteria for exhaustion disorder are often in need of a long-term sick-leave period and the period of impaired ability, while at work, is even longer due to the extreme exhaustion and cognitive problems that these patients experience. Successful prevention is thus of great importance and this is true particularly of demands at work and factors related to leadership issues. However, it is equally important to discuss the contribution of social factors and how society can best act to support individuals such as single parents and those caring for family members or couples with relational conflicts, as these factors most commonly reported can clearly contribute to the development of stress-related problems that require a long and costly treatment and recovery period.

## Competing interests

All of the authors declare that they have no competing interests.

## Authors’ contributions

KH carried out the main part of qualitative and quantitative analysis and drafting of the manuscript. SE participated in the design of the study and helped to draft the manuscript. IJ participated in the design of the study and drafted the manuscript. KS contributed to conception and design of the study, carried out the qualitative analysis in the first qualitative step and ensured validity in the quantitative step, and drafted the manuscript. All authors read and approved the final manuscript.

## Pre-publication history

The pre-publication history for this paper can be accessed here:

http://www.biomedcentral.com/1471-244X/14/66/prepub
